# Acid-Tolerant Photosensitizer: Photodynamic Inactivation of Porphyromonas gingivalis by 2′,4′,5′,7′-Tetraiodofluorescein

**DOI:** 10.3390/pathogens15060567

**Published:** 2026-05-25

**Authors:** Zixiang Wang, Qianwen Deng, Ziyu Huang, Zixing Lin, Haohui Zhu, Janak L. Pathak, Ying Wang, Min Nie

**Affiliations:** 1 Guangdong Engineering Research Center of Oral Restoration and Reconstruction & Guangzhou Key Laboratory of Basic and Applied Research of Oral Regenerative Medicine, Department of Basic Oral Medicine, School of Stomatology, Guangzhou Medical University, Guangzhou 510182, China; 2022119069@stu.gzhmu.edu.cn (Z.W.); 2022119091@stu.gzhmu.edu.cn (Q.D.); 2022119002@stu.gzhmu.edu.cn (Z.H.); 2022119085@stu.gzhmu.edu.cn (Z.L.); haohuizhu@stu.gzhmu.edu.cn (H.Z.); 2 Center of Photosensitive Chemicals Engineering, Feringa Nobel Prize Scientist Joint Research Center, Institute of Fine Chemicals, Frontiers Science Center for Materiobiology and Dynamic Chemistry, School of Chemistry and Molecular Engineering, East China University of Science and Technology, Shanghai 200237, China; 3 Guangdong Engineering Research Center of Oral Restoration and Reconstruction & Guangzhou Key Laboratory of Basic and Applied Research of Oral Regenerative Medicine, Department of General Dentistry Center, School of Stomatology, Guangzhou Medical University, Guangzhou 510182, China

**Keywords:** photodynamic therapy, *Porphyromonas gingivalis*, 2′,4′,5′,7′-tetraiodofluorescein, reactive oxygen species

## Abstract

*Porphyromonas gingivalis* (*P. gingivalis*) is a primary pathogen in periodontitis, yet its elimination is limited by complex anatomical structures. Photodynamic therapy (PDT) is a promising adjunct, but its antimicrobial efficacy is compromised in the acidic microenvironment induced by *P. gingivalis*. Given that 2′,4′,5′,7′-tetraiodofluorescein (TIF) exhibits robust and stable photodynamic activity under acidic conditions, this study investigated the antibacterial effect of TIF-mediated PDT (TIF-PDT) against *P. gingivalis*. *P. gingivalis* ATCC 33277 was treated with TIF (5, 10, 20, and 40 μM), light (525 nm), or both. Reactive oxygen species (ROS) generation was assessed at pH 4.5 or 7.4. Bacterial viability and membrane integrity were evaluated by colony-forming unit (CFU) assay and LIVE/DEAD staining. CFU assays demonstrated that TIF-PDT groups achieved an approximately 4-log reduction in bacterial viability compared to the DEMI, Light, and TIF groups, with no dark cytotoxicity and light-alone effects. LIVE/DEAD staining revealed bright yellow fluorescence in the TIF-PDT (40 μM), indicating membrane damage and significantly lower survival rates than controls. TIF-PDT at 10, 20, and 40 μM produced ROS under both neutral and acidic conditions, exhibited low dark cytotoxicity, and demonstrated potent antibacterial activity against *P. gingivalis* in vitro, suggesting its potential as an acid-tolerant photosensitizer for periodontitis adjunctive therapy.

## 1. Introduction

*Porphyromonas gingivalis* (*P. gingivalis*) is considered a primary pathogen in periodontitis [1], a multifactorial inflammatory condition that chronically leads to the progressive destruction of the periodontal supporting tissues, ultimately resulting in tooth loss and reduced quality of life [1,2,3]. Within dysregulated dental plaque biofilms, the key initiator of periodontitis, *P. gingivalis*, plays a critical role in coordinating microbial dysbiosis and disrupting the host immune response [1]. Therefore, effective elimination of this bacterium has become a crucial target in periodontitis management.

Scaling and root planing, an effective treatment for periodontitis, disrupts the subgingival biofilm and removes dental calculus [2]. Nonetheless, complete biofilm elimination remains challenging in deep periodontal pockets, furcation lesions, and areas with complex root anatomy [3,4,5]. Given that scaling and root planing is time-consuming and technically complicated, incomplete debridement may leave residual bacteria, leading to rapid biofilm regeneration and disease recurrence [4,6].

Photodynamic therapy (PDT) has emerged as a promising adjunctive treatment for periodontitis [2,7]. Activated by specific wavelengths of light, photosensitizers generate reactive oxygen species (ROS) via type I or type II photosensitization, thereby causing non-specific oxidative damage to microbial cell membranes, proteins, and nucleic acids [8,9,10]. The advantages of PDT include minimal invasiveness, avoidance of microbial resistance, neutralization of virulence factors, and the ability to reach deeper regions of periodontal pockets [4,11]. To date, PDT has shown efficacy against *P. gingivalis* both in vitro and in vivo [11,12,13,14].

Nevertheless, the antimicrobial performance of PDT varies with pH [15,16,17]: in acidic microenvironments, limited bactericidal effects are often observed due to aggregation-induced fluorescence quenching and reduced ROS generation [17,18,19,20]. During periodontitis, *P. gingivalis* produces organic acids that reduce local pH, thereby creating an acidic microenvironment in periodontal tissues, which, in turn, initiates and exacerbates the disease [21,22]. Accordingly, photosensitizers for periodontitis treatment must exhibit robust and stable antibacterial activity at low pH.

2′,4′,5′,7′-Tetraiodofluorescein (TIF) is a food additive approved by the US Food and Drug Administration (FDA), European Union, and Chinese National Standard. It demonstrates robust and stable photodynamic bacterial inactivation under neutral and even acidic conditions (pH < 2.0) through halogenation-mediated heavy atom effects and effectively inhibits the growth of multiple bacterial species and *Candida* [23]. Its broad pH adaptability, strong ROS-generating capacity, and established safety as a food additive may enable potent bactericidal activity against *P. gingivalis* in the acidic periodontal environment. Thus, this study aimed to investigate the antibacterial effect of TIF-mediated PDT (TIF-PDT) on *P. gingivalis,* thereby providing a theoretical basis for the utilization of TIF as a novel acid-tolerant photosensitizer to enhance periodontitis treatment.

## 2. Materials and Methods

### 2.1. Photosensitizers

TIF was purchased from Aladdin (Shanghai, China). TIF was dissolved in dimethylsulfoxide (DMSO) to obtain a 20 mM stock solution, which was then stored at 4 °C in the dark. Working solutions of TIF were prepared by serial dilution of the stock solution with sterile distilled water to achieve final concentrations of 5, 10, 20, and 40 μM at room temperature. The stock solution was first diluted to 40 μM, which was subsequently serially diluted twofold to obtain 20, 10, and 5 μM working solutions. All working solutions were used immediately after preparation (within 10 min) to prevent early breakdown.

### 2.2. Absorption Spectra of TIF

Absorbance spectra of TIF under 5, 10, 20, and 40 μM in the water/DMSO mixed solution were recorded before irradiation using a UV–vis spectrophotometer (UV-2600, Shimadzu Corporation, Kyoto, Japan) with a 10 mm × 10 mm quartz glass cuvette over a wavelength range of 200–800 nm at a medium scan rate, with a slit width of 2 nm and a data interval of 1 nm. The cuvette was cleaned with 75% ethanol and rinsed with distilled water prior to each measurement. The baseline was corrected using the same solvent as a blank. All measurements were performed at room temperature.

### 2.3. ROS Generation During TIF-PDT

ROS generation was measured according to the manufacturer’s protocols. For singlet oxygen (^1^O_2_) detection under neutral conditions, 9,10-anthracenediyl-bis(methylene)bismalonic acid (ABDA) (200 μg/mL, Titan Scientific, Shanghai, China) was introduced into varying concentrations of TIF in phosphate-buffered saline (PBS, pH 7.4) [24]. For total ROS detection (hydroxyl radicals •OH, superoxide anions •O_2_^−^, and singlet oxygen ^1^O_2_) under neutral conditions, 3,3′,5,5′-tetramethylbenzidine (TMB) (20 μM, Bepharm Science & Technology, Shanghai, China) was introduced into varying concentrations of TIF in acetic acid–sodium acetate (pH 4.5) [25]. A UV–vis spectrophotometer (UV-2600, Shimadzu Corporation, Kyoto, Japan) was used to measure absorbance at 380 nm for ABDA before and after 0, 20, and 40 s of irradiation [26] and at 650 nm for TMB before and after 5 s of irradiation [27].

### 2.4. Microorganisms and Culture Conditions

*P. gingivalis* strain ATCC 33277 from the State Key Laboratory of Oral Diseases, West China Hospital of Stomatology, National Clinical Research Center for Oral Diseases, Sichuan University, Chengdu, China, was cultured anaerobically (80% N_2_, 10% H_2_, 10% CO_2_; anaerobic workbench AW200SG; Electrotek, West Yorkshire, UK) at 37 °C for 48 h in 14 mL round-bottom tubes (Biofil, Guangzhou, China) with brain heart infusion medium (BHI; Macklin, Shanghai, China) supplemented with K_2_HPO_4_ (76 mM), KH_2_PO_4_ (15 mM), yeast extract (0.3% (*w*/*v*)), vitamins (nicotinic acid (0.04 mM), pyridoxine HCl (0.1 mM), pantothenic acid (0.01 mM), riboflavin (1 μM), thiamine HCl (0.3 μM), and D-biotin (0.05 μM)), amino acids (L-glutamic acid (4 mM), L-arginine (1 mM), L-cysteine HCl (1.3 mM), and L-tryptophan (0.1 mM)), (NH_4_)_2_SO_4_ (10 mM), NaCl (35 mM), and MgSO_4_·7H_2_O (2 mM). The second generation of the bacteria was used for subsequent experiments.

### 2.5. Photodynamic Inactivation

The second passage (48 h) of *P. gingivalis* was transferred to two 24-well plates containing 0, 5, 10, 20, and 40 μM of TIF solutions and incubated in the dark at room temperature for 1 min. The pH of all TIF solutions and bacterial mixtures was measured before irradiation using pH test strips (Macklin, Shanghai, China) and ranged from 6 to 7. One 24-well plate was irradiated with a green LED light (525 nm, 38 mW/cm^2^, 2.28 J/cm^2^) for 1 min, while the other plate was placed in the dark under identical conditions for 1 min.

### 2.6. Bacterial Viability Detection

Colony-forming unit (CFU) assays were performed in triplicate for each experimental condition, as described in Table 1. The pH of all TIF solutions and bacterial mixtures was measured after irradiation using pH test strips (Macklin, Shanghai, China) and ranged from 6 to 7. A volume of 100 μL from each sample, either original or after serial dilution, was applied to Columbia blood agar plates (Huankai, Guangzhou, China). After anaerobic incubation at 37 °C for 120 h, colonies were enumerated as CFU.

### 2.7. Bacterial Membrane Integrity Detection

A LIVE/DEAD bacterial staining kit (DMAO and PI) from Beyotime (Shanghai, China) was used to detect bacterial membrane integrity with undiluted samples. Fluorescence microscopy (OLYMPUS BX43F, OLYMPUS, Tokyo, Japan) was used to examine the stained samples. Green fluorescence was observed in viable bacteria with intact cell membranes, whereas non-viable bacteria with disrupted membranes fluoresced yellow. Image J (1.54r) was used to calculate fluorescence intensities.

### 2.8. Statistical Analysis

SPSS 26.0 (IBM Corporation, Armonk, NY, USA) was employed for statistical analysis. CFU counts were log10-transformed before statistical analysis. Normality of the log10-transformed data was assessed using the Shapiro–Wilk test and Kolmogorov–Smirnov tests and confirmed to follow a normal distribution. As Levene’s test indicated heteroscedasticity (*p* < 0.05), Welch’s analysis of variance was applied for intergroup comparisons, and Tamhane’s T2 test was used for post-hoc multiple comparisons. The survival rates in the LIVE/DEAD bacterial staining assay were compared using an independent *t*-test.

## 3. Results

### 3.1. Absorption Spectra of TIF

**Figure 1:**
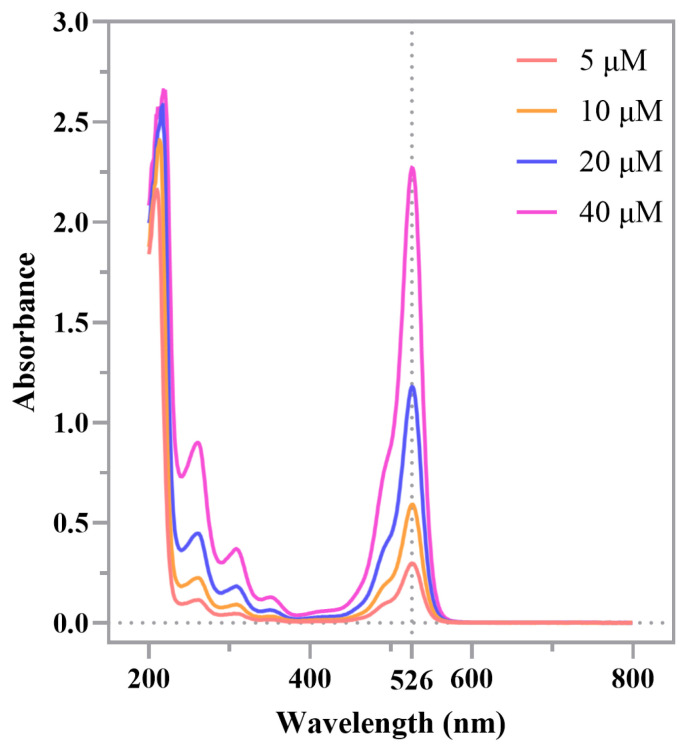
Absorption spectra of TIF at 5, 10, 20, and 40 μM before irradiation. TIF: 2′,4′,5′,7′-Tetraiodofluorescein.

The absorption spectra of TIF at concentrations of 5, 10, 20, and 40 μM were recorded before irradiation. Two maximum absorption peaks were observed at 218 nm and 526 nm. The absorbance in the 450–550 nm range increased progressively with increasing TIF concentration, confirming that TIF can be effectively activated by green light in a concentration-dependent manner (Figure 1).

### 3.2. Reactive Oxygen Species Generation Under Neutral and Acidic Conditions

The ability of TIF to generate ROS under both neutral (pH 7.4) and acidic (pH 4.5) conditions was assessed using two complementary assays.

**Figure 2:**
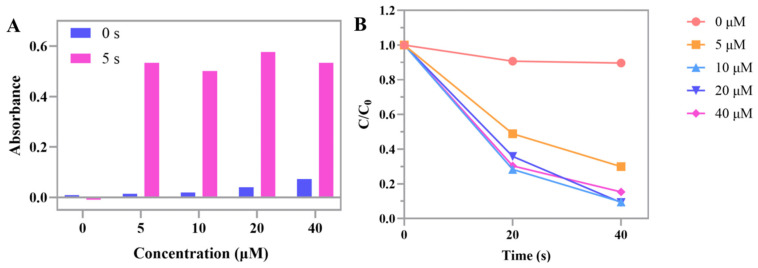
Measurement of reactive oxygen species production by TMB (**A**) and ABDA (**B**) under different concentrations of TIF. TMB: 3,3′,5,5′-tetramethylbenzidine; ABDA: 9,10-anthracenediyl-bis(methylene)bismalonic acid; TIF: 2′,4′,5′,7′-tetraiodofluorescein.

**ROS generation under acidic pH:** The TMB assay (Figure 2A) revealed that under acidic pH (4.5), each TIF concentration (5, 10, 20, and 40 μM) produced detectable levels of total ROS, including hydroxyl radicals (•OH), superoxide anions (•O_2_^−^), and singlet oxygen (^1^O_2_). Notably, the production of these species did not follow a simple concentration-dependent relationship. Among the tested concentrations, 20 μM TIF generated the highest total ROS, while 40 μM TIF produced less ROS than 20 μM, suggesting possible self-quenching or aggregation at higher concentrations.

**ROS generation under neutral pH:** The ABDA assay (Figure 2B) demonstrated that under neutral conditions (pH 7.4), all TIF concentrations produced quantifiable levels of singlet oxygen (^1^O_2_). Again, no linear concentration dependence was observed. The amount of ^1^O_2_ generated by 5 μM TIF was lower than that of the other concentrations. The highest ^1^O_2_ production was observed at 10 μM and 20 μM TIF, whereas 40 μM TIF produced less ^1^O_2_ than these two intermediate concentrations.

These results collectively indicate that TIF retains its photosensitizing capacity even in an acidic environment, a key feature for potential application in periodontitis, where local pH is lowered by bacterial metabolism. Notably, the observation that 40 μM TIF produced lower ROS than 20 μM under both acidic and neutral conditions strongly suggests molecular self-quenching or aggregation at elevated photosensitizer concentrations, a well-recognized phenomenon that reduces singlet oxygen yield.

### 3.3. Photodynamic Inactivation of P. gingivalis Assessed by CFU Assay

The antibacterial efficacy of TIF-PDT against *P. gingivalis* was evaluated using CFU assays under different treatment conditions. The experimental groups included control (DEMI, sterile distilled water without light), light (525 nm irradiation with no TIF), TIF (5, 10, 20, 40 μM without light), and TIF-PDT (same TIF concentrations with light) groups.

**Figure 3:**
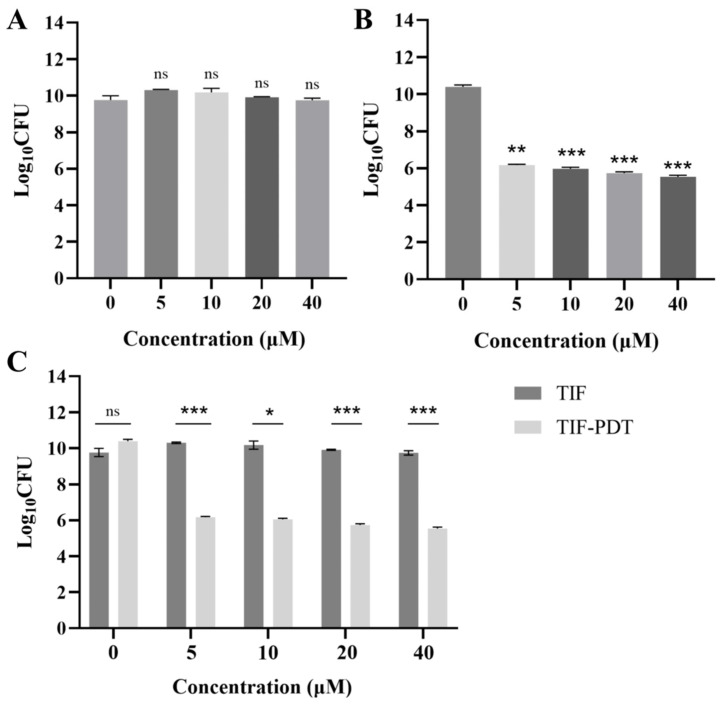
CFU assay of *P. gingivalis* under different concentrations of TIF (5, 10, 20, and 40 μM; light irradiance: 38 mW/cm^2^; irradiation time: 60 s). (**A**) Comparison of different concentrations without irradiation. (**B**) Comparison of different concentrations after irradiation. (**C**) Comparison between TIF-PDT groups and corresponding TIF groups under different concentrations. TIF: 2′,4′,5′,7′-Tetraiodofluorescein. Note. * *p* < 0.05, ** *p* < 0.01, *** *p* < 0.001; ns, not significant.

**Absence of dark cytotoxicity and light-alone effect:** No significant differences in bacterial viability were observed between the DEMI control group and either the light group or any of the TIF groups (Figure 3A,C). This confirms that TIF lacks appreciable dark cytotoxicity and that 525 nm light alone exerts no bactericidal activity against *P. gingivalis*.

**Potent photodynamic bactericidal effect:** In contrast, all TIF-PDT groups exhibited a substantial reduction in bacterial viability, achieving an approximately 4-log reduction compared to the DEMI control group (*p* < 0.05), the light-alone group (*p* < 0.005), and their corresponding TIF-only groups (*p* < 0.05 to *p* < 0.001, Figure 3B,C). This dramatic decrease in CFU counts demonstrates a strong photodynamic effect.

**Concentration trend:** Among the TIF-PDT groups, no statistically significant differences were observed between the different TIF concentrations (5, 10, 20, and 40 μM). However, a clear trend toward lower bacterial viability was observed with higher TIF concentrations (Figure 3B), suggesting that although all tested concentrations are effective, higher concentrations may further enhance killing.

### 3.4. Bacterial Membrane Integrity Assessed by LIVE/DEAD Staining

The membrane integrity of *P. gingivalis* following TIF-PDT was further examined using LIVE/DEAD bacterial staining (DMAO & PI). In this assay, viable bacteria with intact membranes emit green fluorescence, whereas non-viable bacteria with compromised membranes emit both green and red, appearing yellow.

**Figure 4:**
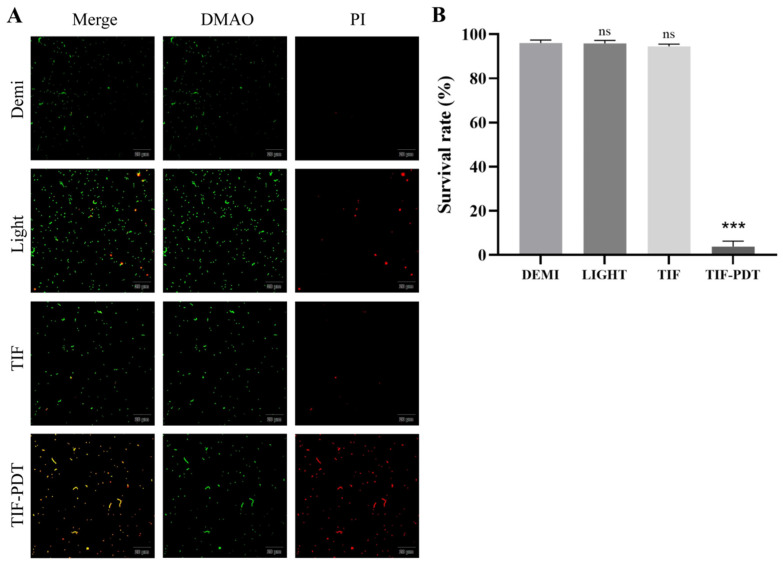
LIVE/DEAD bacteria staining of *P. gingivalis* treated under different conditions (**A**) (scale bar: 20 μm; concentration of TIF: 40 μM; light irradiance: 38 mW/cm^2^; irradiation time: 60 s) and survival rates per group (**B**). At least three separate images for each group were used. TIF: 2′,4′,5′,7′-Tetraiodofluorescein. Note. *** *p* < 0.001; ns, not significant.

**Visual observation:** As shown in Figure 4A, yellow fluorescence (indicative of membrane damage) was negligible in the DEMI control group, the light group, and the TIF (40 μM) group. In sharp contrast, the TIF-PDT (40 μM) group displayed bright yellow fluorescence, indicating extensive membrane disruption.

**Quantitative analysis:** Quantitative analysis of fluorescence intensities (Figure 4B) confirmed that the survival rates of *P. gingivalis* in the TIF-PDT (40 μM) group were significantly lower than those in the DEMI, light, and TIF groups (*p* < 0.001). These results corroborate the CFU findings and further demonstrate that TIF-PDT kills *P. gingivalis* primarily through damage to the bacterial cell membrane.

## 4. Discussion

This in vitro study assessed the absorption spectra and ROS generation of TIF under neutral and acidic conditions. It also demonstrated the photodynamic inactivation of TIF against *P. gingivalis* at different concentrations via CFU assay and LIVE/DEAD bacterial staining. A green LED at 525 nm was selected as a safe and efficient light source for TIF-PDT activation, as ultraviolet light (218 nm) can cause DNA damage [28]. Under irradiation at this wavelength, TIF generated a certain amount of ROS under both acidic and neutral conditions and resulted in an approximately 4-log reduction in CFU of *P. gingivalis*. It was also confirmed that TIF lacked dark cytotoxicity and that 525 nm light alone exerted no bactericidal effect. Notably, although a trend was observed, the bactericidal efficacy of TIF-PDT was not significantly correlated with the tested TIF concentrations.

According to the Chinese National Standard GB 2760-2011, 57 μM is specified as the maximum TIF concentration for use as a food additive. Accordingly, concentrations within the food-safe range (5, 10, 20, and 40 μM) were selected to initially verify and investigate their capacity for ROS generation and photodynamic bactericidal efficacy against *P. gingivalis*.

The ROS production of TIF under both neutral (pH 7.4) and acidic (pH 4.5) conditions was confirmed in this study. The involvement of the type II photosensitization process, characterized by the production of singlet oxygen (^1^O_2_) [29], was also corroborated by the ABDA assay. These findings are consistent with previous studies [23]. Notably, across the tested concentrations (5, 10, 20, and 40 μM), ROS production of TIF did not follow a concentration-dependent pattern: at both neutral and acidic pH, 40 μM TIF generated less ROS than 20 μM TIF. The lack of a clear, concentration-dependent effect of photosensitizers on ROS generation has also been documented in other studies [30]. This observation can be rationalized through the following photophysical mechanisms. First, photosensitizers are prone to form dimers or higher-order aggregates via quenching mechanisms such as π–π stacking and intramolecular charge transfer. Such aggregation reduces the proportion of photoactive monomers, and the aggregated species exhibit a significantly lower singlet oxygen quantum yield compared to their monomeric counterparts [31,32]. Consequently, as the concentration increases, the rising abundance of TIF aggregates may lead to a non-linear or even decreasing trend in ROS production. Meanwhile, higher photosensitizer concentrations can promote triplet-state self-quenching and the formation of transient charge-transfer complexes, both of which reduce singlet oxygen yield by shortening triplet lifetime or inhibiting energy transfer to molecular oxygen [33,34]. Together, these mechanisms offer a plausible explanation for why a simple positive concentration dependence of ROS production was not observed for TIF in the present study. Nevertheless, ROS production at TIF concentrations above 40 μM requires further examination to validate this conclusion.

In periodontitis, *P. gingivalis* metabolizes glucose to generate organic acids that lower the pH of the local periodontal environment [21]. This acidified environment impairs the integrity of the gingival epithelium, contributes to attachment loss, and promotes the progress of inflammation [22,35]. Thus, the stable ROS generation of TIF under acidic conditions enables it to maintain consistent photodynamic bactericidal efficacy within the complex pH environment of periodontitis, demonstrating its potential as an adjunctive therapy.

TIF showed significant antimicrobial efficacy against *P. gingivalis*, with low and non-significant dark cytotoxicity on bacterial cells in this study. This aligns with the essential characteristics of photosensitizers used in PDT [36] and underscores the therapeutic potential of TIF for periodontitis. In accordance with CFU assay results, the TIF-PDT groups achieved an approximately 4-log reduction in bacterial viability compared to both the DEMI and light groups and their corresponding TIF groups. These results corroborate a potent photodynamic bactericidal effect of TIF against *P. gingivalis*. Furthermore, the absence of significant differences between the DEMI group and either the light group or any of the TIF groups indicates that TIF lacks appreciable dark cytotoxicity and that 525 nm light alone exerts no significant bactericidal activity on *P. gingivalis*. This agrees with a previous study that reported a similar conclusion regarding TIF-PDT against *Lactobacillus plantarum* [23]. Notably, the absence of significant differences in CFU results among the tested TIF concentrations for TIF-PDT against *P. gingivalis* is consistent with the comparable ROS levels generated by these concentrations. Given that ROS generation of TIF-PDT was not simply concentration-dependent due to the photophysical mechanisms discussed above, the corresponding lack of concentration-dependent bactericidal activity is mechanistically consistent, though higher concentrations remain to be tested. Overall, ROS detection and CFU assays showed that TIF at 10, 20, and 40 μM generated ROS under both neutral and acidic conditions and exhibited significant photodynamic bactericidal activity against *P. gingivalis.* Consequently, these concentrations possess potential anti-*P. gingivalis* activity in periodontitis therapy.

LIVE/DEAD bacterial staining (DMAO & PI) further verifies the antimicrobial effects and weak dark cytotoxicity of TIF on *P. gingivalis*. Viable and membrane-intact bacteria produced green fluorescence, whereas non-viable bacteria with compromised membranes fluoresced both green and red, appearing yellow [37,38,39]. The yellow fluorescence observed in the TIF-PDT group reflects its antimicrobial efficacy and indirectly corroborates the membrane-damaging bactericidal mechanism of PDT. The absence of significant differences in membrane integrity among the TIF, DEMI, and light groups further demonstrates that TIF lacks dark cytotoxicity and that 525 nm light alone exerts no bactericidal effect. Nevertheless, given the technical limitations of staining methods [40], interference from extracellular nucleic acids [41], and possible viable but non-culturable state of *P. gingivalis* [42], the results may not directly reflect bacterial viability.

Several limitations warrant consideration. Firstly, only a single-species planktonic suspension of *P. gingivalis* was used to evaluate the photodynamic antibacterial efficacy of TIF. Deep periodontal pockets contain multiple species of oral microorganisms coexisting within dental plaque biofilm, where bacteria are more tolerant to antimicrobial treatment due to limited diffusion and biofilm matrix protection [43]. Thus, the current model may not predict efficacy in complex biofilms. Future studies should assess TIF against *P. gingivalis* biofilms and further validate its effects in relevant in vivo periodontitis models. Furthermore, ROS production and bactericidal activity against *P. gingivalis* have not been assessed at higher TIF concentrations, leaving it unclear whether concentration-dependent effects emerge at elevated levels. In addition, all experiments were conducted under neutral pH conditions (pH 6–7). However, the microenvironment of periodontal pockets can be acidic due to inflammation and metabolism of *P. gingivalis* [21,22]. Therefore, the photodynamic efficiency of TIF may be pH-sensitive, and the absence of data under acidic conditions limits the generalizability of our findings. Future studies should evaluate the antibacterial activity of TIF-PDT across a broader pH range relevant to the oral cavity.

As a food additive authorized by multiple countries, TIF demonstrated favorable biocompatibility, as evidenced by the absence of detectable dark cytotoxicity. Furthermore, its steady ROS generation under both acidic and neutral conditions, as well as its efficient photodynamic inactivation against *P. gingivalis*, supports its translational feasibility as an adjunctive therapy for periodontitis. Nonetheless, further investigations are required in multiple respects. For instance, future studies could evaluate the bactericidal efficacy of TIF-PDT under a broader range of TIF concentrations on *P. gingivalis* biofilms, and even on multi-species biofilms derived from periodontitis patients, to provide a more comprehensive theoretical basis for its application in periodontitis treatment.

## 5. Conclusions

TIF-PDT at 10, 20, and 40 μM TIF generated ROS under both neutral and acidic conditions, exhibited low dark cytotoxicity, and demonstrated significant photodynamic antibacterial activity against *P. gingivalis* in vitro, suggesting its potential as an adjunctive therapy for periodontitis.

## Figures and Tables

**Table 1:** Experimental conditions of each group.

Groups	Conditions
Demi	No light; sterile distilled water (control group)
Light	Light; sterile distilled water
TIF 5, 10, 20, and 40 μM	No light; TIF (dark cytotoxicity)
TIF-PDT 5, 10, 20, and 40 μM	Light; TIF (TIF-PDT groups)

## Data Availability

The data presented in this study are available upon request from the corresponding authors.

## Abbreviations

*P. gingivalis*	*Porphyromonas gingivalis*
PDT	Photodynamic therapy
TIF	2′,4′,5′,7′-Tetraiodofluorescein
ROS	Reactive oxygen species
CFU	Colony-forming unit
ABDA	9,10-anthracenediyl-bis(methylene)bismalonic acid
TMB	3,3′,5,5′-tetramethylbenzidine
